# Advances in understanding epigenetic regulation of plant trichome development: a comprehensive review

**DOI:** 10.1093/hr/uhad145

**Published:** 2023-07-19

**Authors:** Yuming Dong, Sen Li, Haoying Wu, Yiming Gao, Zhongxuan Feng, Xi Zhao, Li Shan, Zhongren Zhang, Huazhong Ren, Xingwang Liu

**Affiliations:** College of Horticulture, China Agricultural University, Beijing 100193, China; College of Horticulture, China Agricultural University, Beijing 100193, China; College of Horticulture, China Agricultural University, Beijing 100193, China; College of Horticulture, China Agricultural University, Beijing 100193, China; College of Horticulture, China Agricultural University, Beijing 100193, China; College of Horticulture, China Agricultural University, Beijing 100193, China; College of Horticulture, China Agricultural University, Beijing 100193, China; College of Horticulture, China Agricultural University, Beijing 100193, China; College of Horticulture, China Agricultural University, Beijing 100193, China; Sanya Institute of China Agricultural University, Sanya Hainan 572000, China; College of Horticulture, China Agricultural University, Beijing 100193, China; Sanya Institute of China Agricultural University, Sanya Hainan 572000, China

## Abstract

Plant growth and development are controlled by a complex gene regulatory network, which is currently a focal point of research. It has been established that epigenetic factors play a crucial role in plant growth. Trichomes, specialized appendages that arise from epidermal cells, are of great significance in plant growth and development. As a model system for studying plant development, trichomes possess both commercial and research value. Epigenetic regulation has only recently been implicated in the development of trichomes in a limited number of studies, and microRNA-mediated post-transcriptional regulation appears to dominate in this context. In light of this, we have conducted a review that explores the interplay between epigenetic regulations and the formation of plant trichomes, building upon existing knowledge of hormones and transcription factors in trichome development. Through this review, we aim to deepen our understanding of the regulatory mechanisms underlying trichome formation and shed light on future avenues of research in the field of epigenetics as it pertains to epidermal hair growth.

## Introduction

The plant trichome is a specialized accessory structure that covers aboveground organs and is differentiated from epidermal cells [1]. Trichomes can be classified into unicellular or multicellular types based on their cell composition, and glandular or non-glandular types depending on their secretory function [2]. The morphology and structure of trichomes exhibit significant diversity among different species, and interestingly, even within the same species, different types of trichomes can be found. The size and density of trichomes also vary depending on their location and developmental stage [3]. These variations provide a theoretical foundation for studying the biological functional diversity of trichomes [4].

**Figure 1:**
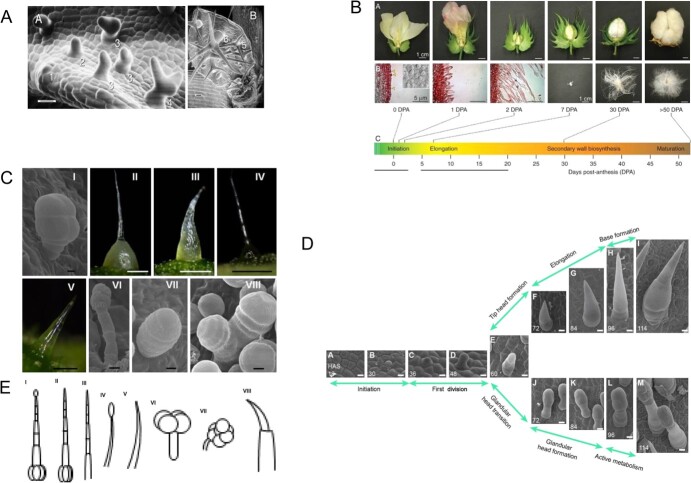
Morphology of plant trichomes [5, 6, 9, 11, 14, 15]. (A) Morphology of *Arabidopsis* trichomes at various developmental stages. Numbers indicate different stages of development [5, 14]. (B) Morphology of cotton fibers at different stages of development [6]. (C) Eight morphological classifications of cucumber trichomes [9]. (D) Five developmental stages of cucumber trichomes [15]. (E) Eight morphological classifications of tomato trichomes [11].

*Arabidopsis thaliana* and *Gossypium hirsutum* (cotton) are representative examples of unicellular trichome plants. *Arabidopsis* trichomes are three-branched, non-glandular and relatively simple in structure (Fig. 1A) [5]. In contrast, cotton fibers are long and unbranched unicellular trichomes (Fig. 1B) [6]. *Cucumis sativus* (cucumber) and *Solanum lycopersicum* (tomato) are multicellular trichome plants that exhibit a great diversity of trichome types [7, 8]. Cucumber trichomes can be categorized into eight types, all of which are multicellular. The majority are type I glandular trichomes and type II non-glandular trichomes [9]. Type I trichomes have a rod-shaped stem of three or four cells and a gland head composed of four or five cells. Type II non-glandular trichomes consist of a multicellular base and a rod-like head composed of three to eight cells [9, 10]. The other types of cucumber trichomes are non-glandular, with the exception of type I and type VI (Fig. 1C) [9]. Tomato epidermal hairs can also be classified into eight types according to their morphological structure. Among them, type II, III, V, and VIII trichomes lack secretory functions and are composed of base and neck cells. Type I, IV, VI, and VII epidermal hairs possess a glandular tip, distinguishing them from the other non-glandular epidermal hairs (Fig. 1E) [11]. Trichomes have distinct morphologies compared with surrounding epidermal cells, making their structure easily observable. Thus, trichomes have become an ideal system for studying the regulation of fate identification, cell differentiation and polarity [12].

The developmental processes of trichomes vary across species due to differences in their morphology. In *Arabidopsis* and cotton, trichome development is divided into four stages. In *Arabidopsis*, trichome growth involves pattern formation, endoreduplication, branch formation, and directional growth (Fig. 1A) [13, 14]. On the other hand, cotton fiber development consists of four sequential and continuous stages, namely initiation, elongation, secondary cell wall (SCW) deposition, and maturity (Fig. 1B) [6]. The development processes of multicellular trichomes are more complex, and as a result studies on these processes have only been reported recently. Cucumber trichomes have five development stages: (i) initiation, (ii) first division, (iii) tip head formation/glandular head transition, (iv) elongation/glandular head formation, and (v) base formation/active metabolism. The third stage is where differentiation of glandular and non-glandular trichomes occurs (Fig. 1D) [15]. However, there is still no clear stage division of tomato epidermal hairs.

Plant trichomes hold significant biological importance and economic value, although they are not essential for the survival of plants. As a barrier between plants and the external environment, trichomes provide protection against various biotic and abiotic stresses [16]. The physical structure of non-glandular trichomes and the chemicals secreted by the glandular trichomes play a role in defending against herbivorous insects [17]. Glandular trichomes possess the ability to synthesize, store, and secrete a wide range of compounds, including proteins, polysaccharides, polyphenols, alkaloids, organic acids, and terpenes. These compounds find applications in the production of spices, essential oils, medical drugs, pesticides, and food additives [18]. Moreover, some specific trichomes, such as cotton fibers, hold considerable economic value. In the case of cucumber fruits, the trichomes, known as fruit spines, significantly influence the market value of commercial fruits, thereby holding important economic significance.

Trichome initiation occurs prior to plant organogenesis, and the initiation and growth of trichomes are controlled through the coordinated actions of plant hormones. Salicylic acid and jasmonic acid (JA) can affect the establishment of trichomes [19]. Overexpression of the *Torenia fournieri* gibberellin 20-oxidase gene (*TfGA20OX2*) increases trichome size and number in *Artemisia annua* [20]. The exploration of key genes has contributed to a gradual enrichment of our understanding of the molecular mechanisms underlying trichome formation. However, there remains a significant research gap, particularly when it comes to comparing different species. Our current knowledge of the regulatory mechanism is more comprehensive for unicellular trichomes compared with multicellular trichomes. The regulatory model of multicellular trichome development involves only a limited number of transcription factors [21]. Research efforts have mainly focused on hormonal and transcriptional regulation, leaving numerous gaps to be filled in our understanding of trichome formation.

In addition to hormones and transcriptional control, epigenetic modifications play a crucial role in plant growth and development [22]. However, a limited number of epigenetic regulators have been reported to regulate the initiation and growth of trichomes. Most of the identified regulators have focused on RNA post-transcriptional modification and microRNA (miRNA)-mediated regulation. Therefore, building upon a brief summary of hormonal and transcriptional regulation, we present a review of the current research progress on epigenetic factors that regulate trichome development in some model plants. Furthermore, we outline potential future research directions in this area. This comprehensive investigation aims to contribute to a deeper understanding of the regulatory mechanisms underlying trichome formation.

## RNA post-transcriptional modification and miRNA regulation

Epigenetic regulations encompass a diverse range of factors, including non-coding RNAs, histone modifications, RNA/DNA methylation and chromatin remodeling, which collectively contribute to the transcriptional/translational regulation of RNA, DNA, and chromatin [23]. Research on plant trichomes has progressively delved into the realm of epigenetics, with particular emphasis on RNA methylation and non-coding RNAs.

### RNA methylation

**Figure 2:**
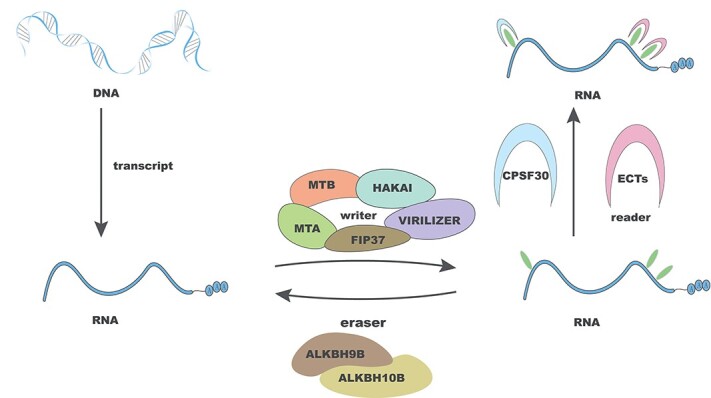
m^6^A modification in plants involves three primary regulatory elements: the reader, writer, and eraser. These elements play pivotal roles in modulating the fate of RNA by introducing, eliminating, and binding m^6^A sites on RNA. Writers, including MTA, MTB, HAKAI, FIP37, and VIRILIZER, function as methyltransferases to add methyl groups to RNA. Erasers, such as ALKBH9B/10B, serve as demethylases for removing methyl groups. Readers, represented by CPSF30 and ECTs, act as recognition factors to identify methylation sites.

Epitranscriptomics studies have led to the discovery of >160 RNA modifications. N6-Adenylate methylation (m^6^A), N1-adenylate methylation (m^1^A) and cytosine hydroxylation (m^5^C) are three types of commonly studied modifications in plant research. m^6^A, the first identified modification, is also the most abundant internal modification of mRNAs [24]. The level of m^6^A and its regulatory role depend on the dynamic interplay among its methyltransferases (writers), demethylases (erasers), and binding proteins (readers) (Fig. 2) [25]. m^6^A methylation modifications play crucial roles in plant growth and stress responses by influencing the translation efficiency and stability of mRNAs. The components of the m^6^A methyltransferase complex, such as MTA, MTB, FIP37, VIRILIZER, and HAKAI, modulate vascular development in *Arabidopsis* [26]. Knockout of FKBP12 INTERACTING PROTEIN 37 KD (FIP37) results in excessive proliferation of the shoot apical meristem in seedlings and delays leaf formation [27]. Additionally, the *fip37-4* mutant exhibits an embryonic lethal phenotype [28]. An RNA demethylase called SlALKBH2 that can bind to *SlDML2* transcripts is required for tomato fruit maturation. Mutation of *SlALKBH2* delays fruit ripening [29]. Deletion of the m^6^A demethylase ALKBH9B reduces the level of viral RNA, restricting the transfer of viral RNA between plant organs, and thereby reducing its systemic infection ability in plants [30]. Additionally, drought stress induces the m^6^A demethylases ZmALKBH10a/b, which can improve the drought resistance of maize [31].

In addition to m^6^A, 5-methylcytosine (m^5^C) is also an important internal mRNA modification. Mutation of tRNA-specific methyltransferase 4B (TRM4B), an RNA m^5^C methyltransferase, leads to a decrease in m^5^C peaks. TRM4B-mediated m^5^C methylation increases the transcript levels of root-related genes, thereby positively regulating root development [32]. In rice, the major m^5^C methyltransferase OsNSUN2 promotes selective translation of certain mRNAs, thereby maintaining chloroplast function and improving heat tolerance [33]. Given its importance in plant growth and stresses response, m^5^C modification likely plays a role in the regulation of trichome development.

### Non-coding RNAs

In eukaryotes, there are certain RNAs that do not undergo protein translation and are referred to as non-coding RNAs (ncRNAs). These ncRNAs play a role in regulating gene expression. Based on their average size, regulatory ncRNAs are classified into long ncRNAs (lncRNAs, >200 nt) and small ncRNAs (sncRNAs, <200 nt) [34]. microRNA is the most prevalent class of endogenous sncRNAs [34]. miRNAs typically range in length from 16 to 29 nt, the majority falling between 20 and 23 nt [35]. The biosynthesis of mature miRNAs is a complex process coordinated by multiple enzymes and auxiliary proteins.

**Figure 3:**
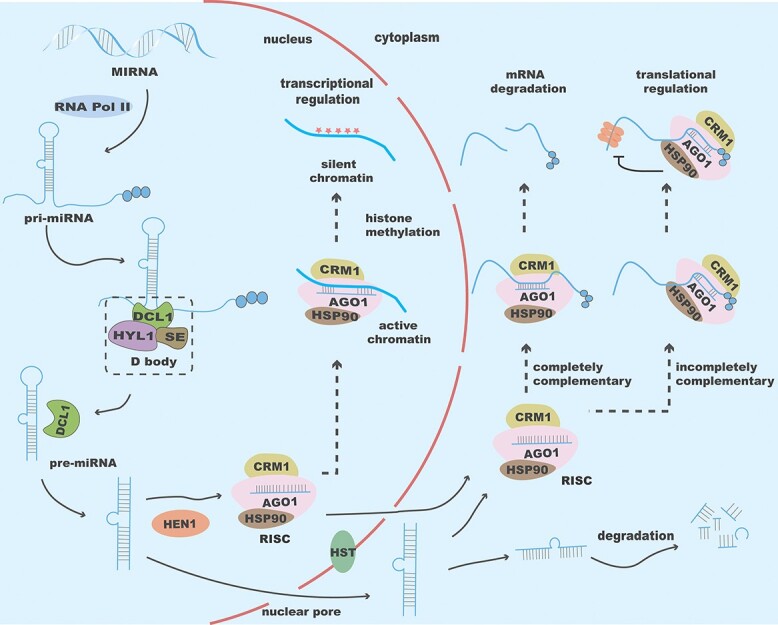
Biosynthesis process and functional mechanism of miRNA. The orange half-arc represents the nuclear membrane. On the left side of the arc, miRNA biosynthesis takes place within the nucleus. miRNA is processed into pri-miRNA by various enzymes in the nucleus, which is further converted into pre-miRNA. The dimer cleaved by DCL1 is processed into a mature miRNA-induced silencing complex (miRISC). Subsequently, miRISC regulates the expression of target genes through three distinct mechanisms.

Plant miRNAs control the expression of target genes during or after transcription. They function by either degrading the target mRNAs through complete complementarity or inhibiting mRNA translation through incomplete complementarity (Fig. 3) [36]. miRNAs have significant impacts on plant growth, morphogenesis, and stress responses [35]. Three members of the miR164 family regulate NAC transcription factors, playing crucial roles in boundary establishment and maintenance, lateral root emergence, vegetative growth, and floral organ formation in *Arabidopsis*[37–39]. Overexpression of sly-miR156 leads to a reduction in fruit quantity and size [40]. In addition, overexpression of miR319b inhibits *TEOSINTE BRANCHED/CYCLOIDEA/PROLIFERATING CELL FACTOR1* (*OsTCP21*), thus reducing the resistance to blast disease in rice [41, 42].

## Regulation of unicellular trichome development

Unicellular trichomes, such as those found on the rosette leaves of *A. thaliana* and the seed coats of *G. hirsutum* (cotton), have been extensively studied, leading to a significant understanding of their developmental mechanism. Researchers have identified numerous transcription factors and plant hormone-related genes that play crucial roles in this process. Additionally, miRNAs have emerged as important participants by inhibiting the expression of these genes post-transcriptionally.

### Trichome development in *A. thaliana*

**Figure 4:**
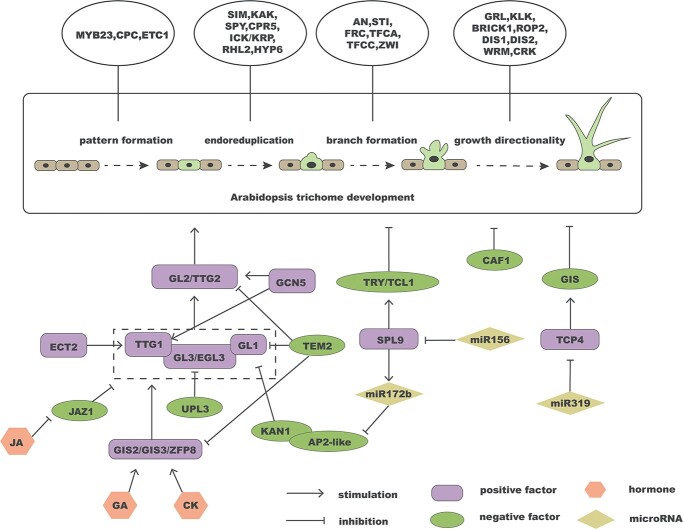
The regulatory network of trichome growth in *A. thaliana*. The rectangular box in the center represents the various stages of trichome formation in *A. thaliana*. The four ovals above the rectangle correspond to every stage of trichome development and contain the genes involved in each stage. The regulatory network for trichome development is shown below the rectangle. The dashed box represents the MYB-bHLH-WD40 (MBW) complex.

Since the 1990s, more than 70 trichome mutants of *A. thaliana* have been discovered [43]. Through the analysis of these mutants and genetic studies, numerous genes have been identified as co-regulators of trichome development. The activation–inhibition model based on *Arabidopsis* trichome development is widely recognized. In this model, the trimeric complex activator MBW is composed of GL1 (the R2R3-MYB transcription factor), TTG1 (WD40 repeat protein), and GL3/EGL3 (bHLH transcription factor). *AtGL1* was the first gene identified, promoting trichome differentiation and development [44]. *TRIPTYCHON* (*TRY*), encoding a small single R3-MYB-repeat protein, was the first negative regulator of trichome development to be reported, lacking an obvious activation domain [45]. The MBW complex facilitates the movement of negative regulators, such as TRY, to neighboring non-epidermal hair cells. In these cells, TRY hinders the formation of the MBW complex, thereby inhibiting the activation of epidermal hairs. The complex also modulates the downstream gene *GL2/TTG2* to control trichome formation [46]. Additionally, numerous other genes have been implicated in controlling trichome formation in *A. thaliana* (Fig. 4).

Plant hormones, such as auxin (IAA), gibberellin (GA), cytokinin (CK), and JA, play important roles in trichome development by regulating downstream genes. C2H2 transcription factors GIS2, GIS3, and ZINC FINGER PROTEIN8 (ZFP8) positively regulate trichome growth via the GA and CK pathways [47, 48]. TEM2 binds to the promoters of *GL1*, *GL2*, *GIS2*, and *ZFP8* to negatively regulate trichome development, and also functions as a link between GA and CK signaling pathways [49]. JASMONATE-ZIM-DOMAIN1 (JAZ1) functions as a repressor of MBW complex formation in the absence of JA by interacting with GL1 and GL3 (the components of the MBW complex). When JA induces JAZ degradation, it promotes the formation of MBW complexes and leads to the development of trichomes (Fig. 4) [50].

One of the most significant epigenetic regulations in plant growth is miRNA-mediated gene silencing, and it also has a very crucial regulatory role in the formation of trichomes. For instance, miR319 modulates trichome branching by inhibiting *PROLIFERATING CELL FACTOR4* (*TCP4*), which promotes the expression of trichome branching inhibitor *GIS* (Fig. 4) [51]. Another important miRNA involved in trichome development is miR156, which targets 10 *SQUAMOSA PROMOTER BINDING PROTEIN LIKE* (*SPL2/3/4/5/6/9/10/11/13/15*) genes involved in the distribution of trichomes during flowering. Among them, SPL9 negatively regulates trichome development independently of the GL1 pathway by directly binding to the promoters of *TCL1* and *TRY* [52]. *MIR156* is expressed during the juvenile growth stage and promotes miR172b transcription via SPL9 (Fig. 4) [53]. The transition from juvenile to adult stages in *A. thaliana* is marked by the appearance of abaxial trichomes on leaves, and it is influenced by two factors: spatial location and time. Spatially, the leaf polarity determinant *KAN1* is expressed exclusively on the abaxial surface of leaves. Temporally, as plants grow, *SPL*s promote the production of miR172 by reducing the inhibitory effect of miR156 on *SPL*s. Consequently, miR172 targets and inhibits AP2-like genes. AP2-like is required to form a complex with KAN1 to inhibit *GL1* gene expression in the distal leaf axis through chromatin loop-mediated histone deacetylation. As plants transition to adulthood, *AP2-like* gradually decreases, relieving this inhibitory effect [54]. Both lateral roots and trichomes are lateral organs, and some transcription factors have opposite effects on their regulation. When the expression of *MIR164* is reduced, the number of lateral roots is increased with the upregulation of *AtNAC1* [39]. miR169 regulates NF-YA transcription factors to affect root growth and branching [55]. Based on the functions of miR164 and miR169 in root development, we speculate that they may also regulate trichome formation.

Post-translational modifications play a crucial role in epigenetics, such as acetylation, ubiquitination, glycosylation, and methylation. They are important regulators of plant growth and participate in regulating trichome development. The m^6^A reader ECT2 regulates trichome morphogenesis by influencing mRNA stability, particularly in 3′ UTR processing. Knockout of ECT2 leads to reduced stabilization of *TTG1*, *ITB1*, and *DIS2* transcripts [56]. The multifunctional histone acetyltransferase GCN5 influences trichome biogenesis by modifying the H3K9/14 acetylation at the promoter regions of core genes such as *GL1*, *GL2*, *GL3*, and *CPC* [57, 58]. Ubiquitin-protein ligase 3 (UPL3) inhibits trichome branching by degrading GL3 and EGL3 [59]. Chromatin assembly factor-1 (CAF-1), a histone chaperone, plays a role in the formation of epidermal hairs via an endonuclease replication-independent pathway. When CAF-1 is lacking, trichomes exhibit supernumerary branches (Fig. 4) [60].

### Development of cotton fibers

**Figure 5:**
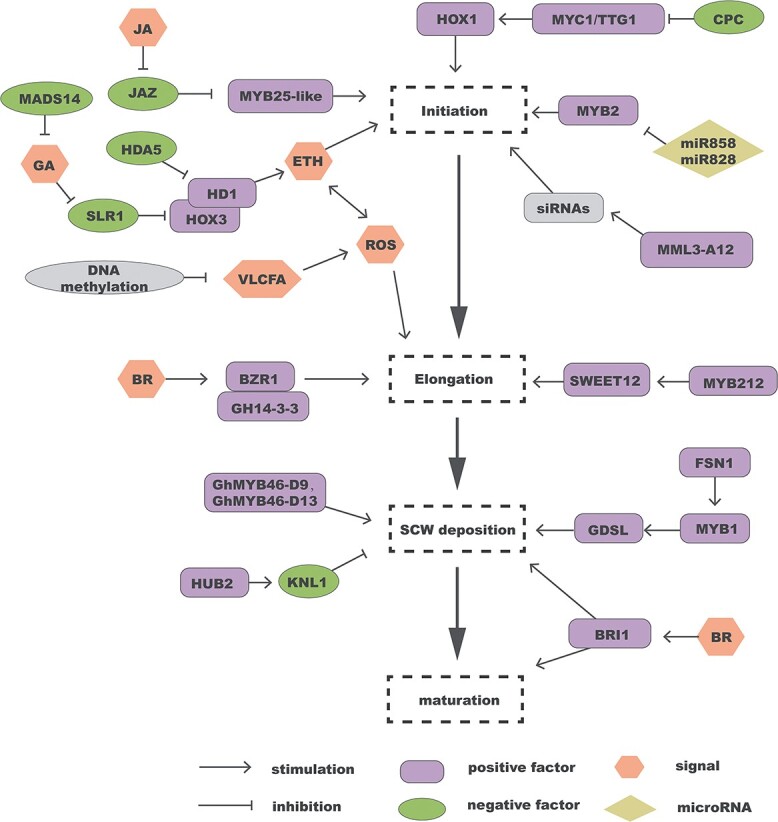
The regulatory network for cotton fiber development. The four dashed rectangles from top to bottom in the middle indicate the four developmental stages of cotton fibers.

There are several similarities between the growth of cotton fibers and *Arabidopsis* trichomes. CPC interacts with MYC1 and TTG1 to negatively regulate cotton fiber initiation [61], while *HOX1* and *HOX3* promote cotton fiber initiation and elongation [62, 64]. MYB212 positively regulates *SWEET12* to facilitate the transport of sucrose and glucose from the ovary to the fiber during cotton fiber elongation [64]. Both MYB46 and FSN1 (a NAC transcription factor) are active during fiber SCW deposition [65], whereas the transcriptional repressor KNL1 negatively regulates cotton fiber development by reducing the transcription of SCW-related genes (Fig. 5) [66].

The formation of cotton fibers is significantly influenced by plant hormones like ethylene, JA, GA, and brassinosteroids (BRs) (Fig. 5**)**. GA promotes the degradation of GhSLR1, thereby releasing GhHOX3 to form a transcriptional complex that promotes cotton fiber growth [63]. Ethylene application to ovules *in vitro* enhances fiber elongation by activating genes crucial for cell wall production and loosening, as well as cytoskeleton organization [67]. JAZ2, a repressor in the JA pathway, negatively regulates fiber initiation by inhibiting the downstream gene *MYB25-like* [68]. Under the combined regulation of ethylene and reactive oxygen species (ROS), fibers progress from the initial stage to the elongation phase [13]. BR stimulates the elongation of cotton fibers by promoting the interaction between Gh14-3-3 and BZR1 [69]. Additionally, BR enhances secondary wall thickening and maturation of cotton fibers through BRI1 [70, 71].

*MYB2D* is targeted by ghr-miR828 and ghr-miR858, which enhance fiber initiation (Fig. 5) [72]. Under drought and salt stress, 163 miRNAs targeting 210 unique genes were identified to be involved in cotton fiber growth [73]. ghr-miR156 may promote cotton fiber initiation by downregulating *SPL9* expression, while ghr-miR36 modulates fiber growth by targeting the bZIP transcription factors (Supplementary Data Table S1) [74]. Seven miRNAs related to fiber initiation were identified using high-throughput sequencing combined with bioinformatic analysis, and their target genes were predicted in the wild-type and fiberless mutants (Supplementary Data Table S1) [75]. Several miRNAs exhibit differential expression patterns across the four developmental stages of fibers, indicating their involvement at different stages. During cotton fiber initiation, the expression level of 33 miRNAs differs between the fiberless type and wild type. In the wild type, 26 miRNAs regulate fiber initiation, and 48 miRNAs control secondary cell wall thickening potentially by targeting *MYB*, *ARF*, *LRR*, and 723 other genes [76]. Deep sequencing identified 32 miRNAs with differential expression between cotton leaves and ovaries, and many fiber-related miRNAs have been validated by qRT–PCR. They are involved in initiation and elongation of fibers by targeting downstream genes (Supplementary Data Table S1) [77]. Differential expression of 46 miRNAs was found during fiber elongation, and the target genes of eight miRNAs and a tasiRNA (*trans*-acting small interfering RNA) were experimentally verified to be associated with fiber elongation through different pathways (Supplementary Data Table S1) [78]. Cellulose synthesis is an indispensable part of fiber maturation. At least three miRNA families (miR396/414/782) target cellulose synthesis-related genes, including fiber synthase, fiber protein Fb23, and fiber quinine oxidoreductase, which are important for the development of cotton fibers (Supplementary Data Table S1) [79]. These miRNAs potentially control the development of cotton fibers (Fig. 5**)**.

Apart from miRNAs, other epigenetic components are crucial for fiber formation as well (Fig. 5**)**. For example, the histone deacetylase HDA5 is specifically expressed during cotton fiber initiation, and its silencing results in reductions of fiber initiation and lint yield [80]. The bidirectional transcripts of *MML3_A12* produce siRNAs that inhibit cotton fiber initiation by mediating self-cleavage of *GhMML3_A12* mRNA in N_1_ plants [81]. Genome-wide analysis revealed that DNA methylation increased during fiber development and targeted genes involved in lipid biosynthesis and spatio-temporal modulation of ROS, thereby regulating fiber differentiation [82]. HISTONE MONOUBIQUITINATION2 (HUB2), a histone H2B mononucleotide E3 ligase, is bound by the transcriptional repressor KNL1, which in turn regulates elongation and SCW deposition of fibers through the ubiquitin-26S proteasome pathway [83, 84].

## Regulation of multicellular trichome development

Although the regulatory mechanisms of uni- and multi-cellular trichomes have numerous similarities, there are also notable differences. It remains unclear whether there is a unified model that governs the regulation of multicellular trichomes, and further exploration is required to understand the underlying mechanisms. At present, the network of trichome growth has been extensively studied only in cucumber and tomato. A preliminary model for the regulation of multicellular trichome development was proposed by studying different mutants of cucumber and tomato, which provides a basis for understanding similar processes in other multicellular trichome-bearing plants.

### Development of cucumber trichomes

**Figure 6:**
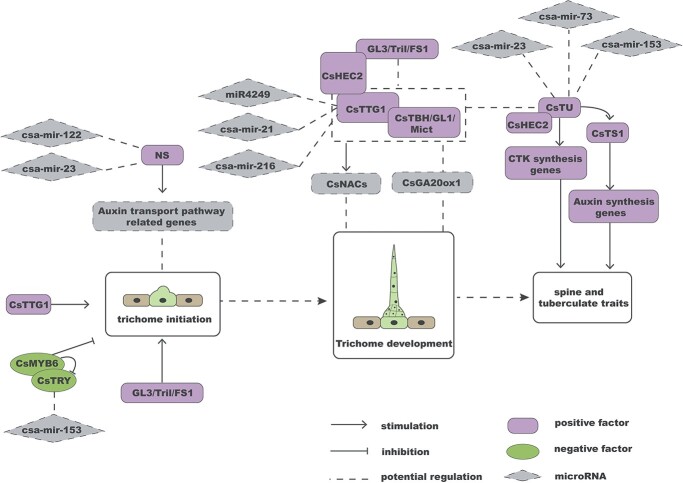
The regulatory network for cucumber trichome development. The miRNAs in the dashed gray diamonds may regulate trichome development by acting on downstream genes. All dashed lines without arrows in the diagram represent only potential regulation.

Considerable research has been conducted on the regulatory mechanisms underlying the formation and growth of cucumber trichomes, particularly fruit spines. Studies have revealed that trichome initiation and development in cucumber are primarily influenced by specific genes and hormones, such as GA, IAA, and CK. Through the interplay of these genes and the crosstalk of plant hormones, a regulatory network of trichome development is proposed (Fig. 6).

The phenotype of a hairless mutant is attributed to the two alleles, *CsGL3* and *Tril*, arising from different mutations of a HD-Zip IV transcription factor (*Csa6M514870*) that controls trichome initiation [85–87]. Positive regulation of trichome development is achieved through the alleles *CsGL1*, *Mict*, and *TBH*, which result from various deletions of *Csa3G748220*. Mutant plants exhibit only cell protrusions on their surfaces, as the complete morphological construction of cucumber trichomes cannot form in *csgl1*, *mict*, and *tbh* mutants [9, 88, 89]. CsMYB6 and CsTRY act as modules to inhibit cucumber trichome initiation [90]. CsTTG1 positively regulates epidermal hair initiation and differentiation by interacting with CsGL1/TBH/Mict [91]. *CsGL1* epistatically acts on *CsTu* to control fruit tubercule formation [92, 93]. Notably, to modulate the density of fruit spines and warts, CsHEC2 directly interacts with CsGL3 and CsTu [94].

Studies have shown that plant hormones such as GA, IAA, and JA regulate the formation of multicellular trichomes. Transcriptome data analysis showed that the GA biosynthetic enzyme gene *CsGA20ox1* was upregulated in *csgl1*. Overexpression of *CsGA20ox1* resulted in shorter trichomes [95]. The auxin transporter-like protein 3 is encoded by a highly conserved *NS* gene in plants, which controls the number of cucumber fruit spines [96]. CsTu directly binds to the promoter of *CsTS1* and synergistically regulates the size of fruit tubercules by affecting auxin content in fruit spines [97]. The expression of *CsNAC*s initially increases and then decreases in response to exogenous GA, IAA, and methyl jasmonate (MeJA). The conserved MeJA-responsive elements (CGTCA), GA-responsive elements (AAACAGA), IAA-responsive elements (AACGAC), and ethylene-responsive elements (ATTTCAAA) are present in the vast majority of *CsNACs* promoters, indicating a link between *CsNAC*s, hormones, and trichome formation [98].

The comprehensive understanding of the impact of epigenetic regulation on cucumber trichomes is still lacking. Analysis of eight publicly available cucumber sRNA-seq databases, coupled with bioinformatic prediction of target genes, resulted in the identification of a limited number of miRNAs targeting several important genes involved in cucumber trichome development in a single database. For instance, csa-miR4249, csa-mir-21, and csa-mir-216 target *CsGL1*, while csa-mir-23, csa-mir-73, and csa-mir-153 target *CsTu*. Furthermore, csa-mir-153 also targets *CsTRY* (Fig. 6) [99]. These findings suggest that miRNAs may play a role in the growth of cucumber trichomes, but their specific contributions have not been definitively established. Currently, there is a lack of molecular experimental evidence to substantiate the significance of miRNAs in cucumber trichome development. An miRNA can target multiple downstream genes, and conversely, a gene can be regulated by multiple miRNAs. Therefore, it requires further investigation whether these miRNAs affect the occurrence and growth of cucumber trichomes through regulating these key genes in the network, along with the underlying molecular mechanisms involved.

### Development of tomato trichomes

**Figure 7:**
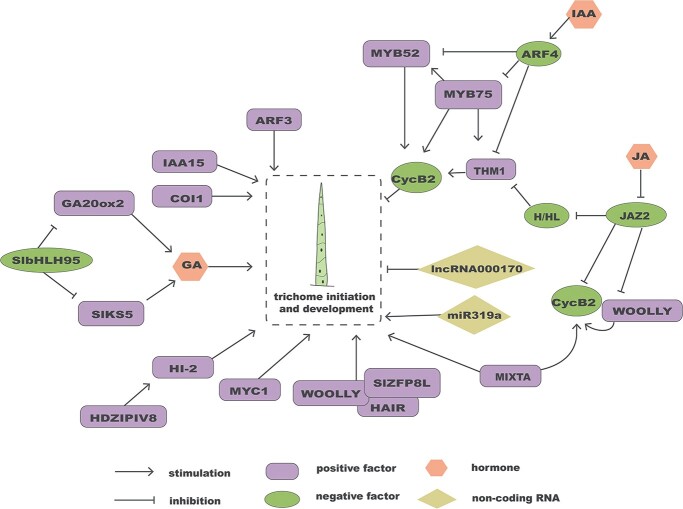
The regulatory network for tomato trichome development. Several important transcription factors and hormones participate in tomato trichome growth.

The regulatory network governing tomato trichomes is relatively straightforward, involving several important transcription factors (Fig. 7). SlMYC1 was reported to be specifically involved in glandular trichome formation. Knockout of *SlMYC1* led to the disappearance of type VI trichomes [100]. *WOOLLY* is the first gene identified during the development of tomato trichomes and is homologous to AtGL2. In the *woolly* mutant, the numbers of both type III and type V trichomes increase, while the number of type IV glandular trichomes decreases. The quantity of type I glandular trichomes increases in *SlWo*-overexpressing plants [101–103]. Overexpression of *SlZFP8-like* (*SlZFP8L*, a close homolog of *Hair*) increases the length and density of epidermal hairs by interacting with WOOLLY [104]. When overexpressing *SlCycB2*, almost all glandular trichomes and non-glandular trichomes (such as types III and V) disappear. Knockdown of *SlCycB2* might stimulate the growth of type III and type V trichomes [105]. SlMIXTA1 (an MYB transcription factor) promotes the production of tomato trichomes [106]. Transcription factors still play a role in regulating the morphology of tomato trichomes. For instance, SlHDZIV8 governs *Hairless-2* expression, which in turn influences trichome shape [107].

The growth of tomato trichomes is controlled by three main hormones: JA, GA, and IAA (Fig. 7). JA regulates the expression of downstream genes *SlCycB2* and *WOOLLY* through JAZ2. Overexpression of *JAZ2* reduces the density of trichomes [108]. *H* and *HL* genes, which are part of the JA signaling pathway, directly repress the expression of the downstream gene *THM1*, which inhibits the development of trichomes [109, 110]. COI1, a critical component of the JA receptor, promotes the occurrence of trichomes [111]. The SlHD8–SlJAZ4 complex plays a significant role in JA-induced elongation of epidermal hairs [112]. bHLH95 regulates GA synthesis by inhibiting *GA20ox2* and *KS5*, thereby regulating the formation of trichomes [113]. The number of tomato type I, V, and VI trichomes decreases in response to downregulation of *SlIAA15* (an auxin family gene) and *SlARF3* (an auxin-responsive factor) [114, 115]. By inhibiting the expression of *THM1* and *MYB52*, ARF4 promotes type II, V, and VI tomato trichome growth [116]. SlMYB75 negatively regulates type II, V, and VI trichomes by affecting several transcriptional pathways [117].

The study of the regulatory network underlying tomato trichome development has primarily focused on transcriptional and hormone regulation, while the role of epigenetic factors in this process has been rarely investigated. Only a few epigenetic regulatory factors have been identified thus far in relation to tomato trichome formation. For instance, overexpression of lncRNA000170 in mature transgenic strains prevents type I trichome production in the lower stem [118]. In addition to slow growth, severe leaf curling, and deepening of leaf fissures, miR319a-overexpressing plants exhibit increased trichome density (Fig. 7). However, which target genes of miR319a contribute to the development of trichomes require further investigation [119].

## Summary and outlook

Trichomes play a crucial role in plants, enhancing their ability to withstand diverse stresses and providing significant economic value. For example, the presence of trichomes on cucumber fruit is an important characteristic that influences consumer preferences. Glandular trichomes, found in many plants, produce secondary metabolites with substantial utility in various industries, including production and medicinal applications.

The regulatory functions of several well-known transcription factors involved in the initiation and morphogenesis of plant trichomes have been partially elucidated. Transcription factor families such as MYB, bHLH, WD40, WRKY, HD-ZIP, NAC, and others, along with hormone-related genes, form the core model for understanding the regulation of plant trichome development. However, the complete and detailed regulatory network is still unknown. The formation of trichomes in plants is modulated by various transcription factors and may be influenced by additional types of transcription factors. Notably, research on multicellular trichomes has progressed at a slower pace compared with that on unicellular trichomes, and many aspects of the regulatory network governing trichome development remain unknown. Therefore, there is ample opportunity to further explore and identify potential regulatory transcription factors for trichome development by leveraging the existing network composed of known genes.

Advancements in molecular biotechnology have greatly contributed to the understanding of epigenetic regulation in various fields, including medicine, microbial metabolism, and plant growth. Epigenetic regulators govern gene expression through multiple pathways and influence nearly all aspects of plant development. In recent years, systematic studies have shed light on the impact of epigenetic modifications on flowering, stress responses, and senescence in different model plants, such as *A. thaliana* and *Oryza sativa*.

As research on plant epigenetic modifications continues to surge, scientists have started to shift their focus towards understanding how epigenetic modifications regulate the development of plant trichomes beyond the scope of transcriptional and hormone regulation. Some epigenetic regulators, such as ETC2, CAF1, GCN5, UPL3, and miR319, were identified as important players in epidermal hair formation in *A. thaliana*. However, only a limited number of miRNAs have been found to be involved in this process. Overall, it remains poorly understood how epigenetic modifications regulate the development of trichomes in plants.

MicroRNAs, although confirmed in a limited number of plant species, have only been studied to a preliminary extent in the context of trichome development. However, the molecular mechanism and regulatory network of these specific miRNAs involved in trichome development are still obscure. Recent advancements in sequencing technologies have rapidly evolved. Small RNA sequencing technology enables in-depth exploration of miRNAs, while degradome sequencing technology efficiently detects the target genes of miRNAs. Differentially expressed miRNAs were identified by analyzing and comparing sRNA-seq databases from wild-type and trichome-defective mutants. Combined with degradome data, the downstream target genes of these miRNAs were further studied to elucidate the molecular mechanism of trichome development.

In addition to interacting with their target genes, miRNAs can also establish connections with other regulators, contributing to a more comprehensive understanding of trichome development. Here are three potential approaches to exploring these interactions. (i) Deciphering the transcriptional regulation of miRNAs by analyzing *cis*-regulatory elements on MIRNA promoters. By identifying the upstream transcription factors of miRNAs that participate in epidermal hair growth, we can expand the TF-miRNA network. (ii) Exploring the role of lncRNAs as endogenous competitive RNAs that indirectly regulate biological processes post-transcriptionally by miRNAs. Incorporating lncRNAs into the network helps establish a large lncRNA-miRNA-gene regulatory network, thereby enriching the regulatory mechanism of trichome formation. (iii) Considering the influence of DNA methylation, which regulates genes involved in lipid biosynthesis and ROS metabolism, thereby influencing cotton fiber development. Given that miRNAs can mediate DNA methylation [120, 121], investigating the regulation of miRNA-regulated DNA methylation in plant trichome formation holds great scientific significance and potential. In conclusion, starting from miRNAs as a focal point will facilitate further breakthroughs in unraveling the mechanisms through which epigenetic factors regulate plant trichome development.

## Supplementary Material

Web_Material_uhad145

## Data Availability

All data supporting the findings of this review are available within the article.

## References

[1] Ma X , Li K, Wang Z et al. Research progress in regulation model in different types of plant trichome. *Sheng Wu Gong Cheng Xue Bao*. 2020;36:2051–65 In Chinese 33169570 10.13345/j.cjb.200114

[2] Feng Z , Bartholomew ES, Liu Z et al. Glandular trichomes: new focus on horticultural crops. *Hortic Res*. 2021;8:158 34193839 10.1038/s41438-021-00592-1 PMC8245418

[3] Andrade MC , Da Silva AA, Neiva IP et al. Inheritance of type IV glandular trichome density and its association with whitefly resistance from *Solanum galapagense* accession LA1401. *Euphytica*. 2017;213:52

[4] Atalay Z , Celep F, Bara F et al. Systematic significance of anatomy and trichome morphology in *Lamium* (Lamioideae; Lamiaceae). *Flora Morphol Distrib Funct Ecol Plants*. 2016;225:60–75

[5] Szymanski DB , Jilk RA, Pollock SM et al. Control of *GL2* expression in *Arabidopsis* leaves and trichomes. *Development*. 1998;125:1161–71 9477315 10.1242/dev.125.7.1161

[6] Basra AS , Malik CP. Development of the cotton fiber. *Int Rev Cytol*. 1984;89:65–113

[7] Zhao JL , Pan JS, Guan Y et al. Transcriptome analysis in *Cucumis sativus* identifies genes involved in multicellular trichome development. Genomics. 2015;105:296–303 25666662 10.1016/j.ygeno.2015.01.010

[8] Zhao JL , Wang YL, Yao DQ et al. Transcriptome profiling of trichome-less reveals genes associated with multicellular trichome development in *Cucumis sativus*. *Mol Gen Genomics*. 2015;290:2007–18 10.1007/s00438-015-1057-z 25952908

[9] Xue S , Dong M, Liu X et al. Classification of fruit trichomes in cucumber and effects of plant hormones on type II fruit trichome development. *Planta*. 2019;249:407–16 30225671 10.1007/s00425-018-3004-9

[10] Chen C , Liu M, Jiang L et al. Transcriptome profiling reveals roles of meristem regulators and polarity genes during fruit trichome development in cucumber (*Cucumis sativus* L.). J Exp Bot. 2014;65:4943–58 24962999 10.1093/jxb/eru258 PMC4144775

[11] Chang J , Xu Z, Li M et al. Spatiotemporal cytoskeleton organizations determine morphogenesis of multicellular trichomes in tomato. *PLoS Genet*. 2019;15:e1008438 31584936 10.1371/journal.pgen.1008438 PMC6812842

[12] Yang C , Ye Z. Trichomes as models for studying plant cell differentiation. *Cell Mol Life Sci*. 2013;70:1937–48 22996257 10.1007/s00018-012-1147-6 PMC11113616

[13] Wang Z , Yang Z, Li F. Updates on molecular mechanisms in the development of branched trichome in *Arabidopsis* and nonbranched in cotton. *Plant Biotechnol J*. 2019;17:1706–22 31111642 10.1111/pbi.13167 PMC6686129

[14] Schellmann S , Hülskamp M. Epidermal differentiation: trichomes in *Arabidopsis* as a model system. *Int J Dev Biol*. 2005;49:579–84 16096966 10.1387/ijdb.051983ss

[15] Dong M , Xue S, Bartholomew ES et al. Transcriptomic and functional analysis provides molecular insights into multicellular trichome development. *Plant Physiol*. 2022;189:301–14 35171294 10.1093/plphys/kiac050 PMC9070826

[16] Huchelmann A , Boutry M, Hachez C. Plant glandular trichomes: natural cell factories of high biotechnological interest. *Plant Physiol*. 2017;175:6–22 28724619 10.1104/pp.17.00727 PMC5580781

[17] Wang Y , Zeng J, Xia X et al. Comparative analysis of leaf trichomes, epidermal wax and defense enzymes activities in response to *Puccinia horiana* in *Chrysanthemum* and *Ajania* species. *Hortic Plant J*. 2020;6:191–8

[18] Chalvin C , Drevensek S, Dron M et al. Genetic control of glandular trichome development. *Trends Plant Sci*. 2020;25:477–87 31983619 10.1016/j.tplants.2019.12.025

[19] Traw MB , Bergelson J. Interactive effects of jasmonic acid, salicylic acid, and gibberellin on induction of trichomes in *Arabidopsis*. *Plant Physiol*. 2003;133:1367–75 14551332 10.1104/pp.103.027086 PMC281631

[20] Inthima P , Nakano M, Otani M et al. Overexpression of the gibberellin 20-oxidase gene from *Torenia fournieri* resulted in modified trichome formation and terpenoid metabolites of *Artemisia annua* L. *Plant Cell Tissue Organ Cult*. 2017;129:223–36

[21] Liu X , Bartholomew E, Cai Y et al. Trichome-related mutants provide a new perspective on multicellular trichome initiation and development in cucumber (*Cucumis sativus* L). *Front Plant Sci*. 2016;7:1187 27559338 10.3389/fpls.2016.01187 PMC4978715

[22] Pikaard CS , Mittelsten SO. Epigenetic regulation in plants. *Cold Spring Harb Perspect Biol*. 2014;6:a019315 25452385 10.1101/cshperspect.a019315 PMC4292151

[23] Tang D , Gallusci P, Lang Z. Fruit development and epigenetic modifications. *New Phytol*. 2020;228:839–44 32506476 10.1111/nph.16724

[24] Liang Z , Riaz A, Chachar S et al. Epigenetic modifications of mRNA and DNA in plants. *Mol Plant*. 2020;13:14–30 31863849 10.1016/j.molp.2019.12.007

[25] Yue J , Wei Y, Zhao M. The reversible methylation of m6A is involved in plant virus infection. *Biology (Basel)*. 2022;11:271 35205137 10.3390/biology11020271 PMC8869485

[26] Růžička K , Zhang M, Campilho A et al. Identification of factors required for m^6^ A mRNA methylation in *Arabidopsis* reveals a role for the conserved E3 ubiquitin ligase HAKAI. *New Phytol*. 2017;215:157–72 28503769 10.1111/nph.14586 PMC5488176

[27] Shen L , Liang Z, Gu X et al. N(6)-Methyladenosine RNA modification regulates shoot stem cell fate in *Arabidopsis*. *Dev Cell*. 2016;38:186–200 27396363 10.1016/j.devcel.2016.06.008 PMC6364302

[28] Vespa L , Vachon G, Berger F et al. The immunophilin-interacting protein AtFIP37 from *Arabidopsis* is essential for plant development and is involved in trichome endoreduplication. *Plant Physiol*. 2004;134:1283–92 15047892 10.1104/pp.103.028050 PMC419804

[29] Zhou L , Tian S, Qin G. RNA methylomes reveal the m^6^A-mediated regulation of DNA demethylase gene *SlDML2* in tomato fruit ripening. *Genome Biol*. 2019;20:156 31387610 10.1186/s13059-019-1771-7 PMC6683476

[30] Martínez-Pérez M , Aparicio F, López-Gresa MP et al. *Arabidopsis* m^6^A demethylase activity modulates viral infection of a plant virus and the m^6^A abundance in its genomic RNAs. *Proc Natl Acad Sci USA*. 2017;114:10755–60 28923956 10.1073/pnas.1703139114 PMC5635872

[31] Miao Z , Zhang T, Qi Y et al. Evolution of the RNA *N^6^*-methyladenosine methylome mediated by genomic duplication. *Plant Physiol*. 2020;182:345–60 31409695 10.1104/pp.19.00323 PMC6945827

[32] Cui X , Liang Z, Shen L et al. 5-Methylcytosine RNA methylation in *Arabidopsis thaliana*. *Mol Plant*. 2017;10:1387–99 28965832 10.1016/j.molp.2017.09.013

[33] Tang Y , Gao CC, Gao Y et al. OsNSUN2-mediated 5-methylcytosine mRNA modification enhances rice adaptation to high temperature. *Dev Cell*. 2020;53:272–286.e7 32275888 10.1016/j.devcel.2020.03.009

[34] Zhang P , Wu W, Chen Q et al. Non-coding RNAs and their integrated networks. *J Integr Bioinform*. 2019;16:20190027 31301674 10.1515/jib-2019-0027 PMC6798851

[35] Carrington JC , Ambros V. Role of microRNAs in plant and animal development. *Science*. 2003;301:336–8 12869753 10.1126/science.1085242

[36] Bartel DP . MicroRNAs: target recognition and regulatory functions. *Cell*. 2009;136:215–33 19167326 10.1016/j.cell.2009.01.002 PMC3794896

[37] Laufs P , Peaucelle A, Morin H et al. MicroRNA regulation of the CUC genes is required for boundary size control in *Arabidopsis* meristems. *Development*. 2004;131:4311–22 15294871 10.1242/dev.01320

[38] Mallory AC , Dugas DV, Bartel DP et al. MicroRNA regulation of NAC-domain targets is required for proper formation and separation of adjacent embryonic, vegetative, and floral organs. *Curr Biol*. 2004;14:1035–46 15202996 10.1016/j.cub.2004.06.022

[39] Guo HS , Xie Q, Fei JF et al. MicroRNA directs mRNA cleavage of the transcription factor *NAC1* to downregulate auxin signals for *Arabidopsis* lateral root development. *Plant Cell*. 2005;17:1376–86 15829603 10.1105/tpc.105.030841 PMC1091761

[40] Zhang X , Zou Z, Zhang J et al. Over-expression of sly-miR156a in tomato results in multiple vegetative and reproductive trait alterations and partial phenocopy of the *sft* mutant. *FEBS Lett*. 2011;585:435–9 21187095 10.1016/j.febslet.2010.12.036

[41] Sunkar R , Zhu JK. Novel and stress-regulated microRNAs and other small RNAs from *Arabidopsis*. *Plant Cell*. 2004;16:2001–19 15258262 10.1105/tpc.104.022830 PMC519194

[42] Zhang X , Bao Y, Shan D et al. *Magnaporthe oryzae* induces the expression of a microRNA to suppress the immune response in rice. Plant Physiol. 2018;177:352–68 29549093 10.1104/pp.17.01665 PMC5933124

[43] Hülskamp M , Misŕa S, Jürgens G. Genetic dissection of trichome cell development in *Arabidopsis*. *Cell*. 1994;76:555–66 8313475 10.1016/0092-8674(94)90118-x

[44] Oppenheimer DG , Herman PL, Sivakumaran S et al. A *myb* gene required for leaf trichome differentiation in *Arabidopsis* is expressed in stipules. *Cell*. 1991;67:483–93 1934056 10.1016/0092-8674(91)90523-2

[45] Schellmann S , Schnittger A, Kirik V et al. *TRIPTYCHON* and *CAPRICE* mediate lateral inhibition during trichome and root hair patterning in *Arabidopsis*. *EMBO J*. 2002;21:5036–46 12356720 10.1093/emboj/cdf524 PMC129046

[46] Han G , Li Y, Qiao Z et al. Advances in the regulation of epidermal cell development by C2H2 zinc finger proteins in plants. *Front Plant Sci*. 2021;12:754512 34630497 10.3389/fpls.2021.754512 PMC8497795

[47] Sun L , Zhang A, Zhou Z et al. *GLABROUS INFLORESCENCE STEMS3* (*GIS3*) regulates trichome initiation and development in *Arabidopsis*. *New Phytol*. 2015;206:220–30 25640859 10.1111/nph.13218

[48] Gan Y , Liu C, Yu H et al. Integration of cytokinin and gibberellin signaling by *Arabidopsis* transcription factors GIS, ZFP8 and GIS2 in the regulation of epidermal cell fate. *Development*. 2007;134:2073–81 17507408 10.1242/dev.005017

[49] Matías-Hernández L , Aguilar-Jaramillo AE, Osnato M et al. TEMPRANILLO reveals the mesophyll as crucial for epidermal trichome formation. *Plant Physiol*. 2016;170:1624–39 26802039 10.1104/pp.15.01309 PMC4775113

[50] Qi T , Song S, Ren Q et al. The jasmonate-ZIM-domain proteins interact with the WD-repeat/bHLH/MYB complexes to regulate jasmonate-mediated anthocyanin accumulation and trichome initiation in *Arabidopsis thaliana*. *Plant Cell*. 2011;23:1795–814 21551388 10.1105/tpc.111.083261 PMC3123955

[51] Vadde BVL , Challa KR, Nath U. The TCP4 transcription factor regulates trichome cell differentiation by directly activating *GLABROUS INFLORESCENCE STEMS* in *Arabidopsis thaliana*. *Plant J*. 2018;93:259–69 29165850 10.1111/tpj.13772

[52] Yu N , Cai WJ, Wang S et al. Temporal control of trichome distribution by microRNA156-targeted *SPL* genes in *Arabidopsis thaliana*. *Plant Cell*. 2010;22:2322–35 20622149 10.1105/tpc.109.072579 PMC2929091

[53] Wu G , Park MY, Conway SR et al. The sequential action of miR156 and miR172 regulates developmental timing in *Arabidopsis*. *Cell*. 2009;138:750–9 19703400 10.1016/j.cell.2009.06.031 PMC2732587

[54] Wang L , Zhou CM, Mai YX et al. A spatiotemporally regulated transcriptional complex underlies heteroblastic development of leaf hairs in *Arabidopsis thaliana*. *EMBO J*. 2019;38:e100063 30842098 10.15252/embj.2018100063 PMC6463210

[55] Sorin C , Declerck M, Christ A et al. A miR169 isoform regulates specific NF-YA targets and root architecture in *Arabidopsis*. New Phytol. 2014;202:1197–211 24533947 10.1111/nph.12735

[56] Wei LH , Song P, Wang Y et al. The m^6^A reader ECT2 controls trichome morphology by affecting mRNA stability in *Arabidopsis*. Plant Cell. 2018;30:968–85 29716990 10.1105/tpc.17.00934 PMC6002187

[57] Kotak J , Saisana M, Gegas V et al. The histone acetyltransferase GCN5 and the transcriptional coactivator ADA2b affect leaf development and trichome morphogenesis in *Arabidopsis*. *Planta*. 2018;248:613–28 29846775 10.1007/s00425-018-2923-9

[58] Wang T , Jia Q, Wang W et al. GCN5 modulates trichome initiation in *Arabidopsis* by manipulating histone acetylation of core trichome initiation regulator genes. *Plant Cell Rep*. 2019;38:755–65 30927071 10.1007/s00299-019-02404-2

[59] Patra B , Pattanaik S, Yuan L. Ubiquitin protein ligase 3 mediates the proteasomal degradation of GLABROUS 3 and ENHANCER OF GLABROUS 3, regulators of trichome development and flavonoid biosynthesis in *Arabidopsis*. *Plant J*. 2013;74:435–47 23373825 10.1111/tpj.12132

[60] Exner V , Gruissem W, Hennig L. Control of trichome branching by chromatin assembly factor-1. *BMC Plant Biol*. 2008;8:54 18477400 10.1186/1471-2229-8-54 PMC2413220

[61] Liu B , Zhu Y, Zhang T. The R3-MYB gene *GhCPC* negatively regulates cotton fiber elongation. *PLoS One*. 2015;10:e0116272 25646816 10.1371/journal.pone.0116272 PMC4315419

[62] Guan XY , Li QJ, Shan CM et al. The HD-zip IV gene *GaHOX1* from cotton is a functional homologue of the *Arabidopsis GLABRA2*. *Physiol Plant*. 2008;134:174–82 18507789 10.1111/j.1399-3054.2008.01115.x

[63] Shan CM , Shangguan XX, Zhao B et al. Control of cotton fibre elongation by a homeodomain transcription factor GhHOX3. *Nat Commun*. 2014;5:5519 25413731 10.1038/ncomms6519 PMC4263147

[64] Zhang J , Huang GQ, Zou D et al. The cotton (*Gossypium hirsutum*) NAC transcription factor (FSN1) as a positive regulator participates in controlling secondary cell wall biosynthesis and modification of fibers. *New Phytol*. 2018;217:625–40 29105766 10.1111/nph.14864

[65] Huang J , Guo Y, Sun Q et al. Genome-wide identification of R2R3-MYB transcription factors regulating secondary cell wall thickening in cotton fiber development. *Plant Cell Physiol*. 2019;60:687–701 30576529 10.1093/pcp/pcy238

[66] Gong SY , Huang GQ, Sun X et al. Cotton *KNL1*, encoding a class II KNOX transcription factor, is involved in regulation of fiber development. *J Exp Bot*. 2014;65:4133–47 24831118 10.1093/jxb/eru182 PMC4112624

[67] Shi YH , Zhu SW, Mao XZ et al. Transcriptome profiling, molecular biological, and physiological studies reveal a major role for ethylene in cotton fiber cell elongation. *Plant Cell*. 2006;18:651–64 16461577 10.1105/tpc.105.040303 PMC1383640

[68] Hu H , He X, Tu L et al. GhJAZ2 negatively regulates cotton fiber initiation by interacting with the R2R3-MYB transcription factor GhMYB25-like. *Plant J*. 2016;88:921–35 27419658 10.1111/tpj.13273

[69] Zhou Y , Zhang ZT, Li M et al. Cotton (*Gossypium hirsutum*) 14-3-3 proteins participate in regulation of fiber initiation and elongation by modulating brassinosteroid signaling. *Plant Biotechnol J*. 2015;13:269–80 25370928 10.1111/pbi.12275

[70] Sun Y , Veerabomma S, Abdel-Mageed HA et al. Brassinosteroid regulates fiber development on cultured cotton ovules. *Plant Cell Physiol*. 2005;46:1384–91 15958497 10.1093/pcp/pci150

[71] Sun Y , Fokar M, Asami T et al. Characterization of the *Brassinosteroid insensitive 1* genes of cotton. *Plant Mol Biol*. 2004;54:221–32 15159624 10.1023/B:PLAN.0000028788.96381.47

[72] Guan X , Pang M, Nah G et al. miR828 and miR858 regulate homoeologous *MYB2* gene functions in *Arabidopsis* trichome and cotton fiber development. *Nat Commun*. 2014;5:3050 24430011 10.1038/ncomms4050

[73] Xie F , Wang Q, Sun R et al. Deep sequencing reveals important roles of microRNAs in response to drought and salinity stress in cotton. *J Exp Bot*. 2015;66:789–804 25371507 10.1093/jxb/eru437 PMC4321542

[74] Zhao T , Xu X, Wang M et al. Identification and profiling of upland cotton microRNAs at fiber initiation stage under exogenous IAA application. *BMC Genomics*. 2019;20:421 31138116 10.1186/s12864-019-5760-8 PMC6537205

[75] Wang ZM , Xue W, Dong CJ et al. A comparative miRNAome analysis reveals seven fiber initiation-related and 36 novel miRNAs in developing cotton ovules. *Mol Plant*. 2012;5:889–900 22138860 10.1093/mp/ssr094

[76] Sun R , Li C, Zhang J et al. Differential expression of microRNAs during fiber development between fuzzless-lintless mutant and its wild-type allotetraploid cotton. *Sci Rep*. 2017;7:3 28127052 10.1038/s41598-017-00038-6 PMC5428375

[77] Xie F , Jones DC, Wang Q et al. Small RNA sequencing identifies miRNA roles in ovule and fiber development. *Plant Biotechnol J*. 2015;13:355–69 25572837 10.1111/pbi.12296

[78] Xue W , Wang Z, Du M et al. Genome-wide analysis of small RNAs reveals eight fiber elongation-related and 257 novel microRNAs in elongating cotton fiber cells. *BMC Genomics*. 2013;14:629 24044642 10.1186/1471-2164-14-629 PMC3849097

[79] Zhang B , Wang Q, Wang K et al. Identification of cotton microRNAs and their targets. *Gene*. 2007;397:26–37 17574351 10.1016/j.gene.2007.03.020

[80] Kumar V , Singh B, Singh SK et al. Role of GhHDA5 in H3K9 deacetylation and fiber initiation in *Gossypium hirsutum*. *Plant J*. 2018;95:1069–83 29952050 10.1111/tpj.14011

[81] Wan Q , Guan X, Yang N et al. Small interfering RNAs from bidirectional transcripts of *GhMML3_A12* regulate cotton fiber development. *New Phytol*. 2016;210:1298–310 26832840 10.1111/nph.13860

[82] Wang M , Wang P, Tu L et al. Multi-omics maps of cotton fibre reveal epigenetic basis for staged single-cell differentiation. *Nucleic Acids Res*. 2016;44:4067–79 27067544 10.1093/nar/gkw238 PMC4872108

[83] Feng H , Li X, Chen H et al. *GhHUB2*, a ubiquitin ligase, is involved in cotton fiber development via the ubiquitin-26S proteasome pathway. *J Exp Bot*. 2018;69:5059–75 30053051 10.1093/jxb/ery269 PMC6184758

[84] Wang Y , Li Y, Gong SY et al. *GhKNL1* controls fiber elongation and secondary cell wall synthesis by repressing its downstream genes in cotton (*Gossypium hirsutum*). *J Integr Plant Biol*. 2022;64:39–55 34796654 10.1111/jipb.13192

[85] Pan Y , Bo K, Cheng Z et al. The loss-of-function *GLABROUS 3* mutation in cucumber is due to LTR-retrotransposon insertion in a class IV HD-ZIP transcription factor gene *CsGL3* that is epistatic over *CsGL1*. *BMC Plant Biol*. 2015;15:302 26714637 10.1186/s12870-015-0693-0 PMC4696102

[86] Cui JY , Miao H, Ding LH et al. A new Glabrous gene (*csgl3*) identified in trichome development in cucumber (*Cucumis sativus* L.). *PLoS One*. 2016;11:e0148422 26845560 10.1371/journal.pone.0148422 PMC4741392

[87] Wang YL , Nie JT, Chen HM et al. Identification and mapping of *Tril*, a homeodomain-leucine zipper gene involved in multicellular trichome initiation in *Cucumis sativus*. *Theor Appl Genet*. 2016;129:305–16 26518574 10.1007/s00122-015-2628-4

[88] Li Q , Cao C, Zhang C et al. The identification of *Cucumis sativus Glabrous 1* (*CsGL1*) required for the formation of trichomes uncovers a novel function for the homeodomain-leucine zipper I gene. *J Exp Bot*. 2015;66:2515–26 25740926 10.1093/jxb/erv046

[89] Zhao JL , Pan JS, Guan Y et al. Micro-trichome as a class I homeodomain-leucine zipper gene regulates multicellular trichome development in *Cucumis sativus*. *J Integr Plant Biol*. 2015;57:925–35 25735194 10.1111/jipb.12345

[90] Yang S , Cai Y, Liu X et al. A *CsMYB6-CsTRY* module regulates fruit trichome initiation in cucumbers. *J Exp Bot*. 2018;69:1887–902 29438529 10.1093/jxb/ery047 PMC6019040

[91] Chen C , Yin S, Liu X et al. The WD-repeat protein CsTTG1 regulates fruit wart formation through interaction with the homeodomain-leucine zipper I protein Mict. *Plant Physiol*. 2016;171:1156–68 27208299 10.1104/pp.16.00112 PMC4902597

[92] Zhang W , He H, Guan Y et al. Identification and mapping of molecular markers linked to the tuberculate fruit gene in the cucumber (*Cucumis sativus* L.). *Theor Appl Genet*. 2010;120:645–54 19847386 10.1007/s00122-009-1182-3

[93] Yang X , Zhang W, He H et al. Tuberculate fruit gene *Tu* encodes a C2H2 zinc finger protein that is required for the warty fruit phenotype in cucumber (*Cucumis sativus* L.). *Plant J*. 2014;78:1034–46 24708549 10.1111/tpj.12531

[94] Wang Z , Wang L, Han L et al. HECATE2 acts with GLABROUS3 and Tu to boost cytokinin biosynthesis and regulate cucumber fruit wart formation. *Plant Physiol*. 2021;187:1619–35 34618075 10.1093/plphys/kiab377 PMC8566225

[95] Sun H , Pang B, Yan J et al. Comprehensive analysis of cucumber gibberellin oxidase family genes and functional characterization of *CsGA20ox1* in root development in *Arabidopsis*. *Int J Mol Sci*. 2018;19:3135 30322023 10.3390/ijms19103135 PMC6213227

[96] Xie Q , Liu P, Shi L et al. Combined fine mapping, genetic diversity, and transcriptome profiling reveals that the auxin transporter gene *ns* plays an important role in cucumber fruit spine development. *Theor Appl Genet*. 2018;131:1239–52 29492617 10.1007/s00122-018-3074-x

[97] Yang S , Wen C, Liu B et al. A *CsTu-TS1* regulatory module promotes fruit tubercule formation in cucumber. *Plant Biotechnol J*. 2019;17:289–301 29905035 10.1111/pbi.12977 PMC6330641

[98] Liu X , Wang T, Bartholomew E et al. Comprehensive analysis of NAC transcription factors and their expression during fruit spine development in cucumber (*Cucumis sativus* L.). *Hortic Res*. 2018;5:31 29872536 10.1038/s41438-018-0036-z PMC5981648

[99] Zhang X , Lai Y, Zhang W et al. MicroRNAs and their targets in cucumber shoot apices in response to temperature and photoperiod. *BMC Genomics*. 2018;19:819 30442111 10.1186/s12864-018-5204-x PMC6238408

[100] Xu J , van Herwijnen ZO, Dräger DB et al. *SlMYC1* regulates type VI glandular trichome formation and terpene biosynthesis in tomato glandular cells. *Plant Cell*. 2018;30:2988–3005 30518626 10.1105/tpc.18.00571 PMC6354261

[101] Yang C , Li H, Zhang J et al. A regulatory gene induces trichome formation and embryo lethality in tomato. Proc Natl Acad Sci USA. 2011;108:11836–41 21730153 10.1073/pnas.1100532108 PMC3141934

[102] Yang C , Li H, Zhang J et al. Fine-mapping of the *woolly* gene controlling multicellular trichome formation and embryonic development in tomato. *Theor Appl Genet*. 2011;123:625–33 21638001 10.1007/s00122-011-1612-x

[103] Hua B , Chang J, Wu M et al. Mediation of JA signalling in glandular trichomes by the *woolly/SlMYC1* regulatory module improves pest resistance in tomato. *Plant Biotechnol J*. 2021;19:375–93 32888338 10.1111/pbi.13473 PMC7868972

[104] Zheng F , Cui L, Li C et al. Hair interacts with SlZFP8-like to regulate the initiation and elongation of trichomes by modulating *SlZFP6* expression in tomato. *J Exp Bot*. 2022;73:228–44 34499170 10.1093/jxb/erab417

[105] Gao S , Gao Y, Xiong C et al. The tomato B-type cyclin gene, *SlCycB2*, plays key roles in reproductive organ development, trichome initiation, terpenoids biosynthesis and *Prodenia litura* defense. *Plant Sci*. 2017;262:103–14 28716406 10.1016/j.plantsci.2017.05.006

[106] Ying S , Su M, Wu Y et al. Trichome regulator *SlMIXTA-like* directly manipulates primary metabolism in tomato fruit. *Plant Biotechnol J*. 2020;18:354–63 31254436 10.1111/pbi.13202 PMC6953195

[107] Xie Q , Gao Y, Li J et al. The HD-zip IV transcription factor *SlHDZIV8* controls multicellular trichome morphology by regulating the expression of *Hairless-2*. *J Exp Bot*. 2020;71:7132–45 32930788 10.1093/jxb/eraa428

[108] Yu X , Chen G, Tang B et al. The jasmonate ZIM-domain protein gene *SlJAZ2* regulates plant morphology and accelerates flower initiation in *Solanum lycopersicum* plants. *Plant Sci*. 2018;267:65–73 29362100 10.1016/j.plantsci.2017.11.008

[109] Hua B , Chang J, Han X et al. H and HL synergistically regulate jasmonate-triggered trichome formation in tomato. *Hortic Res*. 2022;9:uhab080 35048113 10.1093/hr/uhab080 PMC8973001

[110] Chang J , Yu T, Yang Q et al. *Hair*, encoding a single C2H2 zinc-finger protein, regulates multicellular trichome formation in tomato. *Plant J*. 2018;96:90–102 29981215 10.1111/tpj.14018

[111] Thines B , Katsir L, Melotto M et al. JAZ repressor proteins are targets of the SCF(COI1) complex during jasmonate signaling. *Nature*. 2007;448:661–5 17637677 10.1038/nature05960

[112] Hua B , Chang J, Xu Z et al. *HOMEODOMAIN PROTEIN8* mediates jasmonate-triggered trichome elongation in tomato. *New Phytol*. 2021;230:1063–77 33474772 10.1111/nph.17216

[113] Chen Y , Su D, Li J et al. Overexpression of *bHLH95*, a basic helix-loop-helix transcription factor family member, impacts trichome formation via regulating gibberellin biosynthesis in tomato. *J Exp Bot*. 2020;71:3450–62 32133496 10.1093/jxb/eraa114 PMC7475245

[114] Deng W , Yang Y, Ren Z et al. The tomato *SlIAA15* is involved in trichome formation and axillary shoot development. *New Phytol*. 2012;194:379–90 22409484 10.1111/j.1469-8137.2012.04053.x

[115] Zhang X , Yan F, Tang Y et al. Auxin response gene *SlARF3* plays multiple roles in tomato development and is involved in the formation of epidermal cells and trichomes. *Plant Cell Physiol*. 2015;56:2110–24 26412778 10.1093/pcp/pcv136

[116] Yuan Y , Xu X, Luo Y et al. R2R3 MYB-dependent auxin signalling regulates trichome formation, and increased trichome density confers spider mite tolerance on tomato. *Plant Biotechnol J*. 2021;19:138–52 32654333 10.1111/pbi.13448 PMC7769234

[117] Gong Z , Luo Y, Zhang W et al. A *SlMYB75*-centred transcriptional cascade regulates trichome formation and sesquiterpene accumulation in tomato. *J Exp Bot*. 2021;72:3806–20 33619530 10.1093/jxb/erab086

[118] Liao X , Wang J, Zhu S et al. Transcriptomic and functional analyses uncover the regulatory role of lncRNA000170 in tomato multicellular trichome formation. *Plant J*. 2020;104:18–29 32603492 10.1111/tpj.14902

[119] Zilong L , Jingwei F, Rui Y et al. Effects of MiR319a on the growth and development of tomato. *J Beijing Agric Univ*. 2015;30:49–53

[120] Bao N , Lye K, Barton MK. MicroRNA binding sites in *Arabidopsis* class III HD-ZIP mRNAs are required for methylation of the template chromosome. *Dev Cell*. 2004;7:653–62 15525527 10.1016/j.devcel.2004.10.003

[121] Wu L , Zhou H, Zhang J et al. DNA methylation mediated by a microRNA pathway. *Mol Cell*. 2010;38:465–75 20381393 10.1016/j.molcel.2010.03.008

